# The Relationship Between Job-Related Emotional Exhaustion and Frequency of Spiritual Caregiving in Nurses Caring for Elderly Patients: An Example of Turkey

**DOI:** 10.1007/s10943-025-02321-7

**Published:** 2025-04-21

**Authors:** Esra Çavuşoğlu, Abdullah Avcı, Müjde Moran, Meral Gün

**Affiliations:** 1 Department of Medical Nursing, Faculty of Nursing, Mersin University, Çiftlikköy Campus, 33343 Yenişehir, Mersin, Turkey; 2 Mersin University Hospital, Mersin, Turkey

**Keywords:** Elderly patient, Holistic care, Emotional exhaustion, Spiritual care

## Abstract

Exhaustion/burnout, which is often caused by job-related stress among nurses, may have a negative impact on the frequency of spiritual care. The aim of this study was to determine the relationship between job-related emotional exhaustion and the frequency of spiritual care among nurses. A cross-sectional study was conducted with 351 nurses. A negative, moderate to significant relationship was found between the scales used in the study. Our study found that nurses experienced high levels of work-related emotional burnout. It was also found that nurses rarely included spiritual care practices in the care process. We therefore suggest investigating the factors influencing exhaustion and spiritual care in nurses.

## Introduction

Aging is a special stage of life in which physiological, emotional and psychological changes take place at a high level. Physiological changes are particularly pronounced during this period, which can lead to various health problems in elderly people (Ağar, 2020; Brunker et al., 2023). Various processes such as the onset of chronic diseases in older people, the thought of approaching death, loss of role in family and society and being alone can have a negative impact on elderly people’s sense, value judgments and beliefs in life. In this regard, it is very important to support people with a holistic approach to care that incorporates both spiritual processes and physiological characteristics in order to face the changes in the aging process more positively (Acosta & Ely, 2024).

Nursing is a professional healthcare discipline that follows the principle of treating people with a holistic approach to care and recognizes the value of spiritual care (Hawthorne & Gordon, 2020; Ilgaz & Gözüm, 2019). Spiritual care is defined as care based on unconditional love that draws on the individual’s spiritual and cultural beliefs, physical condition, emotions, thoughts and cultural connections, and affirms the unique value of the individual (Aslan et al., 2020; Ramezani et al., 2014). The increased need for physical care in the elderly is more objective and depends on the symptoms, laboratory findings and clinical indicators that occur due to illness. In contrast, however, the presence of spiritual needs is subjective and is usually perceived by the individuals expressing these needs (Jadidi et al., 2021; Lima et al., 2020; Palmer et al., 2018). The literature reports that the need for physical and spiritual care increases in older people as a natural consequence of aging (Lima et al., 2020; Malone & Dadswell, 2018).

It is therefore very important that nurses are aware of the spiritual needs of the elderly people they care for and provide the necessary spiritual care (Aslan et al., 2020; Hawthorne & Gordon, 2020). However, the ability of nurses to provide holistic care at the desired level is closely linked to their own well-being (Hawthorne & Gordon, 2020; Ilgaz & Gözüm, 2019; Jafari & Fallahi-Khoshknab, 2021). Long, tiring working hours, shift work, insomnia and problems in interpersonal relationships can have a negative impact on nurses’ well-being and cause work-related emotional burnout (Harris & Tao, 2022; Rogers et al., 2022; Ruiz-Fernández et al., 2020). Burnout, defined as a state of physical and emotional exhaustion, is a type of mental strain caused by job-related stress. In this context, it is an expected consequence of long-term exposure to a stressful work environment in people-oriented professions such as nursing (Friganović et al., 2019; Sullivan et al., 2022). Emotional burnout can develop over time in nurses who are exposed to chronic individual and environmental stressors in the work environment. This situation can not only reduce nurses’ work performance and motivation, but also negatively affect the duration and frequency of nursing care. Therefore, nurses, who are a sensitive group at high risk of job-related emotional burnout, often focus on physical care and neglect spiritual care. In this sense, recognizing the emotional exhaustion that nurses experience due to their work and the impact of this process on the frequency of spiritual care is extremely important for quality and skilled nursing care (De Diego-Cordero et al., 2022; Harrad & Sulla, 2018; Pérez-Francisco et al., 2020).

In general, the literature search found many different studies in our country and around the world dealing with the concept of burnout and spiritual well-being of nurses. Studies on nurses’ spiritual well-being were also found. However, there are only a limited number of studies looking at the relationship between work-related emotional burnout in nurses and the frequency of spiritual care, similar to our research (Karadag Arli et al., 2017). In particular, the fact that nurses caring for elderly patients are the subject of the study increases the originality of our topic. The process of spiritual care is not really a passive process that only demonstrates the patient’s value judgments, acceptance and spiritual beliefs. It is a dynamic process in which the nurse is involved in the patient’s acceptance and spiritual orientation (Aslan et al., 2020; Nissen et al., 2021). In this sense, our planned research aims to identify the variables that influence the frequency of spiritual caregiving among nurses caring for elderly patients and to make an important contribution to the literature.

## Method

### Sample and Design of the Study

The cross-sectional study was conducted with nurses caring for elderly patients. The study population consisted of 548 nurses working in a university hospital. It was determined that at least 227 nurses should be studied with a 95% confidence interval and a type I margin of error of 0.05 for the sample size (Hespanhol et al., 2019). Nurses who had cared for at least one elderly patient were included in our study, and nurses who worked on neonatal and pediatric wards were not included. Accordingly, 351 nurses who met the inclusion criteria were included in the study.

### Data Collection

The study data were collected between September and November 2022 using the “Personal Information Form, Job-Related Emotional Exhaustion Scale, and Nurse Spiritual Care Therapeutics Scale’”. Data collection was conducted face to face by the researchers. Data collection took approximately 20–30 min for each participant.

*Personal information form*: It was created by the researchers as a result of the literature review (Aslan et al., 2020; Günay, 2021). The form contains seven questions about the nurses’ descriptive characteristics such as age, gender, marital status, presence of children, work experience (year) and field of work.

*Job-Related Emotional Exhaustion Scale*: The scale consists of six items and is unidimensional. The scale was developed by Wharton (1993). The items of the scale were inspired by the Burnout Scale developed by Maslach. The scale is rated on a five-point Likert scale, where 1 = I never feel this way and 5 = I feel this way every day. You can score between 6 and 30 on the scale. The higher the score, the higher the degree of job-related emotional exhaustion. The Turkish validity and reliability study of the scale was conducted by Günay (2021) and the Cronbach’s alpha coefficient was determined to be 0.87 in the said study. In our study, the Cronbach’s alpha coefficient was determined to be 0.94 (Günay, 2021; Wharton, 1993).

*Nurse Spiritual Care Therapeutics Scale*: The scale consists of 17 items and is unidimensional. The scale was developed by Mamier and Taylor (2015), and the Turkish validity and reliability study was conducted by Aslan et al. The scale aims to measure the frequency of nursing interventions or practices aimed at supporting patients’ spirituality. The scale is scored on a five-point Likert scale with 1 (never = 0 times), 2 (rarely = 1–2 times), 3 (sometimes = 3–6 times), 4 (usually = 7–11 times) and 5 (very often = 12 times or more). The scores achievable with the scale range from 17 to 85, with a high score indicating that spiritual care is frequently supported. In their study, Aslan et al. found a Cronbach’s alpha coefficient of 0.86. In our study, the Cronbach’s alpha coefficient was 0.94 (Aslan et al., 2020; Mamier & Taylor, 2015).

### Ethical Approval

Ethics committee (date: 29/08/2022 decision number: 333) and institutional approvals (E-41993462-622.03-2093782) were obtained prior to data collection. In addition, the purpose of the study and the data collection form were explained to the participants and verbal and written consent was obtained from the participants prior to data collection. The study was conducted according to the principles of the Declaration of Helsinki.

### Statistical Evaluation

Numbers, percentages, mean, standard deviation and minimum–maximum values from descriptive statistics were used to analyze the data. In this study, hierarchical regression analysis was used to determine the variables that predict the frequency of nurses providing spiritual care. Categorical variables were included in the hierarchical regression after being converted into dummy variables. Also the enter method was used in the hierarchical regression model. The relationship between the scales used in the study was analyzed using the Pearson correlation test. The significance level for all statistical evaluations was accepted as *p* < 0.05.

## Results

Table 1 shows the descriptive characteristics of the participants according to categorical and continuous variables and the distribution of the scale mean values. Accordingly, it was found that the majority of participants were female, married and childless. In addition, it was found that the majority of the participants had a bachelor’s degree and worked mostly in the wards. It was found that the mean age of the nurses participating in the study was 32.58 ± 6.91 years and the mean work experience (year) was 9.62 ± 7.16 years. In our study, it was found that the mean score of the Job-Related Emotional Exhaustion Scale was 20.43 ± 7.03 and the mean score of the Spiritual Care Therapeutics Scale was 34.19 ± 15.10.

**Table 1:** Characteristics of the participants

Categorical variables (*n* = 351)	*n*	%
*Gender*		
Female	236	67.2
Male	115	32.8
*Marital status*		
Single	173	49.3
Married	178	50.7
*Have a child*		
Yes	174	49.6
No	177	50.4
*Education status*		
High school/Associate	56	16.0
Bachelor	267	76.0
Postgraduate	28	8.0
*Working department*		
Clinic	242	68.9
Intensive care unit	109	31.1
Continuous variables (*n* = 351)	Min–max	mean ± SD
Age	21.0–50.0	32.58 ± 6.91
Work experience (year)	1.0–29.0	9.62 ± 7.16
Job-Related Emotional Exhaustion Scale	6.0–30.0	20.43 ± 7.03
Nurse Spiritual Care Therapeutics Scale	17.0–85.0	34.19 ± 15.10

The participants’ responses to the scale items were examined. In the Job-Related Emotional Exhaustion Scale, it was found that most participants responded to item 2, item 3, item 5 and item 6 with “5 = I feel like this every day”. In the spiritual care therapy scale, it was found that most of the participants responded “1 = never (0 times)” to items 4, 6, 8, 9, 10, 11, 13, 14, 15, 16 and 17 (Table 2).

**Table 2:** Distribution of participants’ answers to scale items (n = 351)

*Job-Related Emotional Exhaustion Scale*					
Items *n* (%)	I never feel this way	I don’t feel this way most of the time	I feel this way sometimes	I feel this way most of the time	I feel this way every day
Item 2 = I feel exhausted at the end of the workday	21 (6.0)	33 (9.4)	83 (23.6)	88 (25.1)	**126 (35.9)**
Item 3 = I’m fed up with getting up in the morning and having to face a new day at work	62 (17.7)	69 (19.6)	87 (24.8)	41 (11.7)	**92 (26.2)**
Item 5 = I feel disappointed with my job	54 (15.4)	63 (17.9)	81 (23.1)	60 (17.1)	**93 (26.5)**
Item 6 = I feel like I work hard at my job	24 (6.8)	44 (12.6)	81 (23.1)	84 (23.9)	**118 (33.6)**

*Nurse Spiritual Care Therapeutics Scale*					
Items *n* (%)	Never	1–2 times	3–6 times	7–11 times	At least 12 times
Item 4 = Frequency of assessment of the patient’s health-related spiritual or religious beliefs or practices	**120 (34.2)**	110 (31.3)	71 (20.2)	22 (6.3)	28 (8.0)
Item 6 = Frequency of encouraging the patient to talk about the influence of God on their illness	**157 (44.7)**	104 (29.6)	53 (15.1)	14 (4.0)	23 (6.6)
Item 8 = Frequency of recording the patient’s spiritual care	**187 (53.3)**	84 (23.9)	43 (12.3)	17 (4.8)	20 (5.7)
Item 9 = Frequency of discussion with colleagues about the spiritual care the patient needs	**146 (41.6)**	110 (31.3)	55 (15.7)	17 (4.8)	23 (6.6)
Item 10 = Frequency with which a counsellor visits the patient	**273 (77.8)**	44 (12.5)	21 (6.0)	2 (0.6)	11 (3.1)
Item 11 = Frequency with which the patient can consult their religious or spiritual counsellor	**268 (76.4)**	43 (12.2)	27 (7.7)	2 (0.6)	11 (3.1)
Item 13 = Frequency of encouraging the patient to talk about the spiritual challenges of living with the disease	**131 (37.3)**	120 (34.2)	58 (16.5)	22 (6.3)	20 (5.7)
Item 14 = Frequency with which you suggest prayer to the patient	**161 (45.9)**	101 (28.8)	57 (16.2)	14 (4.0)	18 (5.1)
Item 15 = Frequency of recommending the reading of a spiritually satisfying passage (such as the patient’s scriptures)	**231 (65.8)**	66 (18.8)	37 (10.5)	3 (0.9)	14 (4.0)
Item 16 = Frequency of mentioning spiritual resources to the patient	**194 (55.3)**	98 (27.9)	42 (11.9)	3 (0.9)	14 (4.0)
Item 17 = Frequency of stay for care after completion of task	**131 (37.3)**	108 (30.8)	64 (18.2)	19 (5.4)	29 (8.3)

In the study, a hierarchical regression analysis was performed to determine the variables predicting the frequency of spiritual care provided by nurses. Accordingly, the independent variables were included in the analysis separately in order of importance and the results (Model 1, Model 2 and Model 3) were analyzed. Model 1 included the extent to which work-related emotional exhaustion predicted the frequency of spiritual care. In the model in question, work-related emotional exhaustion (*B* = − 1.028 *p* < 0.001) was found to explain 22.7% of the frequency of spiritual care (Table 3).

**Table 3:** Predictors of the frequency of spiritual care among nurses caring for elderly patients (*n* = 351)

Model (Enter)	Independent variables	B	SE	Beta	t	p	CI (95%)	
							LB	UB
*Model 1*								
Durbin–Watson = 1.745 *F* = 103.561 ***p* < 0.001** *R* = 0.478 *R*^2^ = 0.229 **Adjusted *R***^**2**^** = 0.227**	(Constant)	55.204	2.182		25.294	** < 0.001**	50.912	59.497
	Job-Related Emotional Exhaustion	− 1.028	0.101	− 0.478	− 10.177	** < 0.001**	− 1.227	− 0.829
*Model 2*								
Durbin–Watson = 1.745 *F* = 40.377 ***p* < 0.001** *R* = 0.509 *R*^2^ = 0.259 **Adjusted *R***^**2**^** = 0.252**	(Constant)	56.180	2.189		25.662	** < 0.001**	51.874	60.485
	Job-Related Emotional Exhaustion	− 0.872	0.108	− 0.406	− 8.044	** < 0.001**	− 1.085	− 0.659
	Working department (ICU)	− 3.409	1.585	− 0.105	− 2.150	**0.032**	− 6.527	− 0.291
	Work experience (year)	− 0.323	0.101	− 0.153	− 3.189	**0.002**	− 0.522	− 0.124
*Model 3*								
Durbin–Watson = 1.745 *F* = 15.740 ***p* < 0.001** *R* = 0.519 *R*^2^ = 0.269 **Adjusted *R***^**2**^** = 0.252**	(Constant)	61.468	7.465		8.234	** < 0.001**	46.785	76.151
	Job-Related Emotional Exhaustion	− 0.826	0.113	− 0.384	− 7.307	** < 0.001**	− 1.049	− 0.604
	Working department (ICU)	− 3.449	1.644	− 0.106	− 2.098	**0.037**	− 6.683	− 0.215
	Work experience (year)	0.010	0.302	0.005	0.033	0.974	− 0.585	0.605
	Gender (Female)	− 2.682	1.673	− 0.083	− 1.603	0.110	− 5.973	0.610
	Age	− 0.242	0.312	− 0.111	− 0.776	0.438	− 0.856	0.372
	Marital status (Married)	0.696	2.199	0.023	0.316	0.752	− 3.629	5.021
	Have a child (Yes)	− 1.776	2.358	− 0.059	− 0.753	0.452	− 6.415	2.863
	Education status (Bachelor & Postgraduate)	0.968	2.200	0.024	0.440	0.660	− 3.359	5.295

Dependent variable: Nurse Spiritual Care Therapeutics Scale. SE, Standard error; CI, confidence interval; LB, lower bound; UB, upper bound; ICU, intensive care unit

Model 2 includes the extent to which work conditions and emotional exhaustion predict the frequency of spiritual care. Accordingly, the resulting model was found to be significant. According to the Model 2, emotional exhaustion (*B* = − 0.872 *p* < 0.001), working department (*B* = − 3.409 *p* = 0.032) and work experience (year) (*B* = − 0.323 *p* = 0.002) explained 25.2% of the frequency of spiritual care. Compared to Model 1, the total variance in Model 2 was found to increase from 22.7 to 25.2% (Table 3).

In Model 3, the effects of emotional exhaustion and working conditions as well as sociodemographic variables (age, gender, marital status, having a child, education status) on the frequency of spiritual care were examined. In this context, it was found that all independent variables in Model 3 explained the frequency of spiritual care by 25.2% and the model obtained was significant. Compared to Model 2, it was found that the independent variables specified in Model 3 did not cause a change in the overall variance ratio (Table 3).

There was a negative, moderate to significant relationship between job-related emotional exhaustion and the spiritual care therapeutics scales used in this study (*r* = − 0.478 *p* < 0.001). Accordingly, it was found that as job-related emotional exhaustion increased, the frequency of spiritual care among nurses decreased (Table 4).

**Table 4:** Correlation between the scales and independent variables (*n* = 351)

Variables (*n* = 351)		Job-Related Emotional Exhaustion Scale	Nurse Spiritual Care Therapeutics Scale
Job-Related Emotional Exhaustion Scale	r	–	− 0.478**
	*p*	–	< 0.001

^**^Correlation is significant at the 0.01 level (two-tailed)

## Discussion

This section discusses the prevalence of work-related emotional exhaustion and spiritual care in nurses caring for elderly patients, in line with the literature. In this study, the incidence of spiritual care by nurses caring for elderly patients was found to be low. A review of the literature found a very limited number of studies similar to our study addressing the frequency of spiritual care and the level of emotional exhaustion of nurses. The study by Karadag Arli et al. reports that the more frequently nurses provide spiritual care, the higher the level of burnout (Karadag Arli et al., 2017). Inadequate working conditions in hospitals and excessive workload of nurses can be considered as one of the reasons for this situation. It is noteworthy that the studies found in the literature review generally focused on nurses’ spiritual well-being and perceptions of spirituality rather than the frequency of spiritual care provided by nurses (Rogers et al., 2022; Seid & Eneyew, 2022; Yıldırım & Ertem, 2022). This situation can be seen as the missing piece of the puzzle in a professional discipline that, like nursing, aims for a holistic approach. This is because the impact of spiritual care, which includes an individual’s own value judgments, spiritual beliefs and acceptance, is also very important to overall well-being. Especially in older patients, factors such as the presence of chronic illnesses, the feeling of being close to death, the decline of roles in family and society and the feeling of loneliness can cause negative changes in the meaning, beliefs and values of life (Britt et al., 2023; Stelcer et al., 2023). This situation can increase the need for spiritual support care in older people. In this sense, it is very important to support elderly patients, who are a particularly vulnerable group, in terms of spiritual care. Considering the gap in the literature, our study focused on the variables that predict the frequency of spiritual care provided by nurses caring for elderly patients.

In this study, the most important variable predicting the frequency of spiritual care provided by nurses was found to be work-related emotional exhaustion. It was found that most of the nurses think that they work hard in their job, feel exhausted at the end of work and most of them are disappointed with their job. In this context, work-related emotional exhaustion was found to be high among the nurses who participated in our study. In the literature review, it was reported that nurses experience moderate to high levels of emotional exhaustion (Mudallal et al., 2017; Poku et al., 2020).

Nursing is a professional health discipline which, due to the nature of the profession, must ensure continuous and optimal care (Cao et al., 2023; Cheraghi et al., 2023). In this sense, given factors such as harsh working conditions, shift work, low wages and an insecure job description, as well as continuous and complex care processes, nurses can be expected to experience high levels of burnout. Although it is to be expected that nurses experience high levels of burnout due to these factors, the saddest thing is that most nurses are disappointed with their job. This is because it is extremely important to love and embrace the work in a profession like nursing, which requires a lot of commitment and this has a strong impact on the quality and type of care provided (Zamanzadeh et al., 2015). In this study, it was found that another factor that influences the frequency of spiritual care provided by nurses is the clinic they work in and the year they work. It was found that nurses, especially those who work in the intensive care unit (ICU) and have more years of experience, are less likely to provide spiritual care. It can be considered a surprising result that nurses working in ICU provide less spiritual care. Given the nature of the critical care environment and the physical and spiritual needs of ICU patients, it is to be expected that spiritual care would play a greater role in the ICU. However, because it is an intense, stressful, dynamic process with high care needs in the ICU environment, nurses may focus on physical care and deemphasize spiritual care. Furthermore, it can be said that nurses working in ICU are unfamiliar with the concept of spirituality or have limited knowledge on the subject. In a study conducted on this topic, it was found that ICU nurses do not have sufficient knowledge about spiritual care (Willemse et al., 2018). This supports our research findings. In the context of our research, it can be said that the problems experienced by nurses with longer working years due to the work process and emotional exhaustion have led to the postponement of spiritual care.

This study found that the frequency of spiritual care was not influenced by sociodemographic characteristics such as gender, age, marital status, children and education level (Aslan et al., 2020; Neathery et al., 2020; Vogel & Schep-Akkerman, 2018). Our research findings are consistent with the literature in this direction.

This study also examined the participants’ responses to the spiritual care scale. Accordingly, it was found that most nurses did not assess patients’ spirituality and spiritual practices and most of them did not talk to their colleagues about patients’ spiritual care needs. The main reasons for this situation are the high burnout level of the nurses, their workload and their exhaustion. In addition, it can be said that nurses are reluctant to adopt a spiritual approach because they do not have sufficient knowledge about spirituality, the need for spiritual care and spiritual practices.

In this study, it was found that there was a negative, moderate to significant relationship between work-related emotional exhaustion and the frequency of spiritual care. Accordingly, it was found that as nurses’ emotional exhaustion increased, the frequency of spiritual care decreased. As we found in our research, this could be due to the high burnout level of nurses and their insufficient knowledge about spirituality and the need for spiritual care.

This research is extremely important in that it deals with two important concepts such as burnout and spiritual care, which are extremely important in a professional profession such as nursing. Holistic care is the foundation of nursing. In this context, the need for spiritual care, which increases with age, should also be considered in holistic nursing. The ability of nurses to provide holistic care at the desired level is closely related to their own well-being. As nurses’ well-being is affected by individual and environmental stressors at work, their level of emotional exhaustion also increases. In this sense, the finding that the level of burnout experienced by nurses influences/reduces the frequency of spiritual care is one of the notable findings of our research. In addition, the literature search found only a limited number of studies that examined the relationship between work-related emotional exhaustion and the frequency of spiritual care (Karadag Arli et al., 2017). In this sense, investigating the burnout level of a special group such as nurses and the frequency of spiritual care is the strength of our research.

## Limitations

The fact that this study was conducted at a single center is the first limitation. Therefore, the results cannot be generalized to the whole population. Another limitation is that there may be communication problems during data collection due to the long, busy working days of the nurses.

## Conclusion

This study revealed that nurses caring for elderly patients experience high levels of work-related burnout. Our study also found that nurses provide low levels of spiritual care. In addition, the most important variable predicting the frequency with which nurses provide spiritual care was found to be work-related emotional exhaustion. In this situation, it is advisable to regularly assess nurses’ burnout levels and consider the personal and environmental variables that influence burnout. In addition, it is advisable to improve caregivers’ knowledge of spirituality and the importance of spiritual care through continuing education.
